# A Leaf Extract of *Harrisonia abyssinica* Ameliorates Neurobehavioral, Histological and Biochemical Changes in the Hippocampus of Rats with Aluminum Chloride-Induced Alzheimer’s Disease

**DOI:** 10.3390/antiox10060947

**Published:** 2021-06-11

**Authors:** Hend Mohamed Anwar, Gehan S. Georgy, Sherin Ramadan Hamad, Wafaa K. Badr, Mohamed A. El Raey, Mohamed A. O. Abdelfattah, Michael Wink, Mansour Sobeh

**Affiliations:** 1 Department of Biochemistry, National Organization for Drug Control and Research, Giza 11221, Egypt; hend.hassan@outlook.de; 2 Department of Pharmacology, National Organization for Drug Control and Research, Giza 11221, Egypt; gehan_gorgy11@hotmail.com; 3 Department of Histopathology, National Organization for Drug Control and Research, Cairo 11221, Egypt; manaa_82@yahoo.com; 4 Department of Medicinal Plants and Natural Products, National Organization of Drug Control and Research, Giza 11221, Egypt; wafaakorany@ymail.com; 5 Phytochemistry and Plant Systematics Department, National Research Centre, Dokki, Giza 12622, Egypt; elraiy@gmail.com; 6 College of Engineering and Technology, American University of the Middle East, Kuwait; mohamed.abdelmoety@aum.edu.kw; 7 Institute of Pharmacy and Molecular Biotechnology, Heidelberg University, Im Neuenheimer Feld 329, 69120 Heidelberg, Germany; wink@uni-heidelberg.de; 8 AgroBioSciences, Mohammed VI Polytechnic University, Lot 660–Hay MoulayRachid, Ben-Guerir 43150, Morocco

**Keywords:** *Harrisonia abyssinica*, hippocampus, Alzheimer’s, polyphenols, ChE, ERK, Bcl2

## Abstract

Aluminum (Al) is an omnipresent mineral element in the environment. The brain is a central target of Al toxicity, being highly susceptible to oxidative damage. Therefore, recognition of drugs or natural products that guard against Al-mediated neuronal cell death is a powerful strategy for prevention and treatment of neurodegenerative disorders. This work aimed to explore the potential of a leaf extract from *Harrisonia abyssinica* to modulate the neurobehavioral, biochemical and histopathological activities induced experimentally by Al in vivo. Rats subjected to Al treatment displayed a reduction in learning and memory performance in a passive avoidance test accompanied by a decrease in the hippocampal monoamine and glutamate levels in addition to suppression of Bcl2 expression. Moreover, malondialdehyde (MDA), inflammatory markers (TNF-α, IL-1β), apoptotic markers (caspase-3 and expression of Bax) and extracellular regulated kinase (ERK1/2) levels were elevated along with acetylcholinesterase (AChE) activity, histological changes and marked deposition of amyloid β plaques in the hippocampus region of the brain tissues being observed in Al-treated animals. Concomitant administration of the high dose of *H. abyssinica* (200 mg/kg b.w.) restored nearly normal levels of all parameters measured, rather than the low dose (100 mg/kg b.w.), an effect that was comparable to the reference drug (rivastigmine). Molecular docking revealed the appropriate potential of the extract components to block the active site of AChE and ERK2. In conclusion, *H. abyssinica* leaf extract conferred neuroprotection against Al-induced neurotoxic effects, most likely due to its high phenolic and flavonoid content.

## 1. Introduction

Alzheimer’s disease (AD) is a chronic multifactorial neurodegenerative disorder that is characterized by chronic neuro-inflammation, extracellular deposition of amyloid beta (Aβ) plaques and formation of neurofibrillary tangles due to tau protein hyper-phosphorylation [1,2]. Such events lead to cholinergic deficits as they affect the morphology and functions of synapses, disrupt the signaling pathways of neurons and destroy dendritic spines, leading to decline in memory, cognition and behavior [3]. On the other hand, oxidative stress induced inflammation is another culprit that aids the development and progression of AD.

A substantial cure for AD that stops or even slows down the damage of neurons is still not available. The currently available remedies just work to relieve the symptoms of the disease. Thus, there is a strong demand for safe, effective and multi-mechanistic therapies to properly manage and control AD. In this regard, natural secondary metabolites could be safe and potent alternatives [4]. 

*Harrisonia abyssinica* (family Rutaceae) is a small tree or shrub native to Eastern, Central and Southern Africa. The plant is widely used in African folk medicine for the treatment of menstrual problems, stomach pains, gonorrhea, skin diseases, fever, dysentery, hemorrhoids, snakebites and tuberculosis [5]. The leaves, roots and stem bark are used in the form of decoction, powder and infusion to treat fever, malaria, diarrhea, migraine, diabetes, urinary problems, general body pain, intestinal worms and as a wash to disinfect wounds and abscesses [5,6]. 

Moreover, extracts from the leaf, bark and root parts of the plant exhibit a wide array of biological activities, including antimicrobial, antifungal, antimalarial, cytotoxic, insect antifeedant, antiviral and molluscicidal activities [5,6].

Several compounds were isolated from the plant parts, such as limonoids, steroids and prenylated polyketides [6,7]. Harrisonin, 12β-acetoxy harrisonin, obacunone, deoxyobacunone, 5-dehydrooriciopsin, pedonin and atalantolide are some of the limonoids detected in the leaves [6,7]. Root extracts were reported to yield the limonoid 11β,12β-diacetoxyharrisonin, the quassinoid perforaquassin A and three chromones; namely, peucenin, alloptaeroxylin and *O*-methylalloptaeroxylin [7]. The cycloterpene cycloabyssinone was isolated from the stem bark [8]. The prenylated acetophenones harronin I and harronin II were isolated from the berries and displayed potent antibacterial and antifungal activities [9]. Moreover, chebulagic acid and chebulanin were isolated from the plant’s roots and exhibited potent antifungal activity in vitro [10]. 

In this work, we profiled the secondary metabolites in a methanol extract from *H. abyssinica* leaves using LC-MS/MS and determined its antioxidant and anti-Alzheimer’s activities in rats with Al-induced AD. The effect of the extract on the levels of several neurotransmitters, biomarkers and proteins associated with AD was also investigated. Additionally, molecular docking of the extract’s major components towards acetylcholinesterase (AChE) and extracellular regulated kinase 2 (ERK2) was performed to evaluate their potential to block these two key target enzymes in AD pathogenesis. 

## 2. Materials and Methods

### 2.1. Plant Material and Extraction

The plant leaves were collected from Lupaga Site in Shinyanga, Tanzania [11]. A voucher specimen was kept under P7305 at IPMB, Heidelberg University. Dried leaves (250 g) were ground and extracted with methanol at room temperature (3 × 1 L). The extracts were filtered, evaporated under vacuum and subjected to freeze-drying to yield 35 g of the dried powder.

### 2.2. HPLC-PDA-ESI-MS/MS

A Thermo Finnigan LCQ-Duo ion trap mass spectrometer (Thermo Fisher Scientific, Waltham, MA, USA) with an ESI source (ThermoQuest Corporation, Austin, TX, USA) was used to identify the phytochemical composition of the leaf extract as previously reported [12]. 

### 2.3. Total Phenolic Content and In Vitro Antioxidant Activities

The total phenolic content, DPPH (2,2,1-diphenyl-1-picrylhydrazyl) radical scavenging assay and FRAP (Ferric Reducing Antioxidant Power) assay were performed as previously described [13,14]. 

### 2.4. Animals

Adult male Wistar albino rats (*Rattus norvegicus*) weighing 100–120 g were used in this study. Rats were obtained from the National Organization for Drug Control and Research (NODCAR), Cairo, Egypt. They were housed in stainless steel cages in a well-ventilated room and fed on a commercially available standard diet. Tap water was given ad libitum. Animals’ proper care and use was maintained under the supervision of the Animal Ethics Committee of National Organization for Drug Control and Research (NODCAR-REC, ID: NODCAR/II/25/2020).

### 2.5. Experimental Design

After two weeks of acclimatization, rats were randomly divided into five groups of ten animals each, as per the following: I. Control group: rats received saline orally. II. Aluminum chloride (AlCl_3_)-treated group: animals received aluminum chloride (AlCl_3_) at a dose of 100 mg/kg b.w./day p.o. [15]. III. AlCl_3_/rivastigmine-treated group: animals received AlCl_3_ at a dose of 100 mg/kg BW p.o. and rivastigmine (0.3 mg/kg, i.p.) for 3 weeks (Mahdy et al. 2014). IV. AlCl_3_/low dose of *H. abyssinica*: animals received the extract at a dose of 100 mg/kg BW/day by oral gavage intragastrically for 3 weeks. V. AlCl_3_/high dose of *H. abyssinica*: animals received the extract at a dose of 200 mg/kg BW/day by oral gavage intragastrically for 3 weeks. The rats were euthanized by cervical dislocation after the behavioral evaluation, their hippocampus was dissected and frozen at −80 °C for the subsequent experimental estimations and two hippocampi from each group were prepared for histological studies using hematoxylin and eosin (H&E) staining.

### 2.6. Behavioral Passive Avoidance Test

The “step-through” apparatus contained two compartments: one illuminated with an electric bulb, 220 V and 40 W, with a top *Plexiglas* cover; and another dark compartment of equal size and a stainless-steel bar floor. The two compartments were separated by a sliding door. The floor of the dark compartment was connected to a constant current stimulator. First, a rat was placed in the illuminated compartment and allowed to explore the entire apparatus for 3 min. On the following day and after the rat entered the dark compartment, the sliding door was shut, and a scrambled electric shock (5 sec, 0.6 mA) was delivered through the grid floor. The latency to avoid the shock-associated compartment as a sign of non-agreeable stimulus memory (retention test) was recorded on two consecutive days [16].

### 2.7. Biochemical Analysis

#### 2.7.1. Quantification of Neurotransmitters in the Hippocampus

Neurotransmitters in the hippocampus were determined by a HPLC system that consisted of a rheodine injector, a column oven, quaternary pump and UV variable wavelength detector. The sample was extracted by 70% methanol (HPLC grade) then injected directly into an AQUA column (150 mm × 5 µ C18, purchased from Phenomenex, Des Plaines, IL, USA) using a mobile phase of potassium phosphate (20 mM, pH 2.7): methanol (70:30) with a flow rate 1.5 mL/min and UV wavelength of 270 nm.

#### 2.7.2. Estimation of TNF-α, MDA, IL-1 Beta and Caspase-3 in the Hippocampus

TNF-alpha, rat IL-1beta and caspase-3 were measured by the colorimetric ELISA kit, Sino Gene Clon and bioassay technology laboratory kits, respectively, using a Biotek ELISA Synergy HTX Multi-Mode Reader. MDA was estimated by a kit purchased from Biodiagnostic (Biodiagnostic, Giza, Egypt). Assays were performed according to the manufacturer’s instructions.

#### 2.7.3. Estimation of Acetylcholine Esterase (AChE) Activity and Glutamate in the Hippocampus

The AChE activity in the hippocampus was determined according to Ellman et al. [17] using DTNB–phosphate after incubating 0.01 mL hippocampus homogenate (the homogenate was prepared in saline using homogenizer mixer) with acetyl thiocholine iodide for 10 min. Rat glutamate was estimated using rat glutamate colorimetric ELISA kit (cat no. MBS756400), assay was achieved as stated by the manufacturer.

#### 2.7.4. Estimation of Extracellular Regulated Kinase (ERK) Level in the Hippocampus

Quantitative detection of ERK level in the hippocampus was performed using an ERK rat ELISA kit (Bioassay Technology Laboratory, Korain Biotech Co., Ltd., E1256Ra, Shanghai, China). Assay was carried out according to the manufacturer’s instructions.

### 2.8. Bax and Bcl2 Gene Expression Using RT-PCR

The last hippocampus tissue portion was stored in RNA lysis solution at −80 °C, allowing for genetic processing. Assessment of Bax and Bcl2 gene expression was done by real-time quantitative reverse transcription PCR (RT-PCR). Total RNA was extracted from frozen samples using TRIzol^®^ reagent (Invitrogen, Sigma-Aldrich, St. Louis, MO, USA) according to a standard protocol. The isolated total RNA was converted into complementary DNA (cDNA) using SMART Scribe™ Reverse Transcriptase (Clontech Laboratories, Inc. A Takara Bio Company, Nojihigashi, Kusatsu, Shiga, Japan). RT-PCR was performed using a Real-Time PCR v 7.9 System (DTlite, DNA technology, LLC, , Moscow, Russia) and SYBR^®^ Green PCR Master Mix (QIAGEN) in a final volume of 25 µL with the following thermal cycling conditions: 95 °C for 15 s, followed by 40 cycles of 95 °C for 15 s, 60 °C for 15 s and 72 °C for 45 s. The sequences of PCR primer pairs used for each gene are shown below (Table 1). Data were analyzed with the ABI Prism sequence detection system software and quantified using the v1⋅7 Sequence Detection Software from PE Biosystems (Foster City, CA, USA). Relative expression of the studied genes was calculated using the comparative threshold cycle method. All values were normalized to the GAPDH gene as an invariant endogenous control (reference gene).

### 2.9. Hematoxylin and Eosin Staining of the Hippocampus

After being fixed for 48 h in 10% formalin, hippocampal tissue was washed and dehydrated in ascending grades of alcohol, cleared in xylene and finally embedded in paraffin wax. For histological analysis, paraffin sections (five-micron thick) were prepared, placed on clean slides and stained with Ehrlich’s hematoxylin–eosin (Bancroft J, Gamble M, 2008).

### 2.10. Congo Red Staining of the Hippocampus

Congo red-stained sections were examined and observed under a digital microscope at 400× magnification.

### 2.11. Molecular Docking

Major compounds identified in *H. abyssinica* leaves extract were docked into the active sites of extracellular regulated kinase 2 (ERK2) and acetylcholinesterase (AChE). The X-ray crystallographic structures of the two enzymes (PDB codes: 5K4I and 4EY7, respectively) were downloaded from the Protein Data Bank (www.rcsb.org). Docking was performed using the Molecular Operating Environment (MOE, 2010.10; Chemical Computing Group Inc., Montreal, QC, Canada). The downloaded proteins were cleared from all repetitive amino acid chains and protonated by adding the hydrogen atoms. Chemical structures of the docked compounds were either downloaded from PubChem or drawn using the MOE builder tool. Ionization states of the compounds at pH 7.0 were considered and partial charges were adjusted accordingly. Energy minimization was performed using the force field MMFF94x on all compounds and the docking protocol adopted the default settings of placement (Triangle Matcher), scoring (London dG, London, UK) and refinement.

### 2.12. Statistical Analysis

Statistical analysis was performed using GraphPad Prism Software Inc, version 6.0. Data are presented as mean ± SE. Levels of significance were accepted with *p* < 0.05. One-way ANOVA was used for multiple comparisons, followed by a Tukey test.

## 3. Results

### 3.1. HPLC-MS/MS and ^1^HNMR

**Figure 1:**
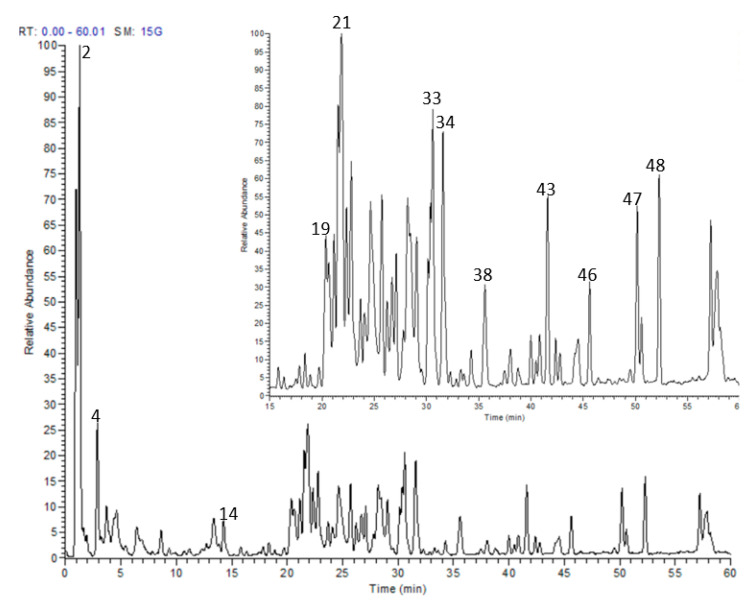
LC-MS profile of *H. abyssinica* leaves methanolic extract.

Altogether, we tentatively identified 49 secondary metabolites in a methanol extract from *H. abyssinica* leaves utilizing LC-MS/MS and ^1^HNMR. Gallotannins, phenolic acids and ellagitannins were shown to prevail in the extract (Figure 1 and Table 2). Theogallin, a galloylquinic acid ester, was characterized based on its [M-H]^−^ at *m*/*z* 343 and two fragment ions 169 and 191, in addition to ^1^HNMR signals at δ ppm 7.02 attributed to galloyl moiety and 5.31 (m) attributed to H-5 of quinic acid moiety, and at δ ppm 1.8 to 2.2 attributed to H-2 and H-6 of quinic acid moiety. Gallic acid, a trihydroxy benzoic acid, was annotated based on its [M-H]^−^ at *m*/*z* 169, the major fragment ion 125 and an ^1^HNMR signal at δ ppm 6.9. Chlorogenic acid was also identified according to its [M-H]^−^ at *m*/*z* 353 and two fragment ions at 179 and 191. It was confirmed by comparison with a reference standard.

Several hydrolysable tannins were recognized in the extract. They demonstrated [M-H]^−^ at *m*/*z* 301, 633, 635, 951 and 953 and they were identified as ellagic acid, galloyl-HHDP-hexoside, 1,2,6-trigalloyl glucose, geraniin and chebulagic acid, respectively. Their structures were confirmed by the presence of a group of broad signals that resonated in the aromatic region at δ ppm 7.39, attributed to the ellagic acid derivatives, and at δ ppm 7.03, 7.02 and 7.01, that were attributed to the galloyl group of galloyl glucoses, corilagin and geraniin. Moreover, a characteristic anomeric proton signal of corilagin resonated at δ ppm 6.24 (d, J = 8 Hz), and hexahydroxy diphenoyl signals resonated from δ ppm 6.3 to 6.6 [18].

Quinic acid was among the major compounds along with other derivatives such as *p*-coumaroylquinic acid. Quinic acid exhibited an [M-H]^−^ at *m*/*z* 191 and three fragment ions at 85, 127 and 173, while *p*-coumaroylquinic acid showed an [M-H]^−^ at *m*/*z* 337 and two product ions at 119 and 163. The ^1^HNMR signals from 1.5 to 3.0 ppm were attributed to the H-2 and H-6 protons of quinic acid and its derivatives. A series of flavonoids were also detected. Their retention times, molecular weights and fragmentation pattern are shown in Table 2. They demonstrated several ^1^HNMR signals around δ ppm 6.2 and 6.4 that are attributed to H-6 and H-8 of flavonoids.

**Table 2:** Analysis of secondary metabolites from *H. abyssinica* leaves utilizing LC-MS/MS.

No.	Rt	[M-H]^−^	MS/MS	Tentatively Identified Compound
1	1.43	191	85, 127, 173	Quinic acid
2	1.63	343	169, 191	Theogallin
3	1.93	169	125	Gallic acid
4	2.97	353	179, 191	Chlorogenic acid
5	3.07	495	169, 343	3,4-di-*O*-galloylquinic acid
6	3.65	337	119, 163	*p*-coumaroylquinic acid
7	4.89	647	343, 495	3,4,5-tri-*O*-galloylquinic acid
8	6.97	477	169, 313, 433	Coumaroyl galloylglucose
9	8.64	183	125, 169, 183	Methylgallate ^a^
10	9.35	301	229, 257, 301	Ellagic acid
11	11.21	953	301, 463, 633, 935	Chebulagic acid ^b^
12	12.73	951	301, 613, 933	Geraniin
13	13.52	633	301, 463	Galloyl-HHDP-hexoside
14	14.62	453	169, 313, 327	Pyrogallol-*O*-methylgalloyl glucose
15	15.85	635	331, 483	1,2,6-trigalloyl glucose
16	17.80	635	313, 465, 483	1,3,6-trigalloyl glucose
17	18.87	537	169, 271, 313	Sinapoyl galloyl glucose
18	19.67	551	169, 271, 533	Gallic acid glucoside derivative
19	20.30	615	301, 463	Quercetin 3-*O*-(6″-O-galloyl) glucoside
20	21.04	449	179, 449	Myricetin 3-*O*- pentoside
21	21.81	615	179, 301, 463	Quercetin 7-(6’’-galloylglucoside)
22	22.37	431	269, 311, 341	Isovitexin
23	23.10	609	179, 301	Rutin
24	24.18	463	179, 255, 301	Quercetin 3-*O*-glucoside
25	23.93	539	169, 271, 313	Gallic acid glucoside derivative
26	24.59	599	169, 285, 447	Kaempferol 3-*O*-(6’’-galloyl) glucopyranoside
27	25.58	433	179, 301	Quercetin 3-*O*-pentoside
28	26.76	433	151, 179, 301	Quercetin 5-*O*-pentoside
29	27.12	447	151, 285	Kaempferol 3-*O*-glucoside
30	28.47	447	151, 285	Kaempferol 5-*O*-glucoside
31	29.04	599	169, 285, 313, 447	Kaempferol galloyl glucoside
32	30.13	417	179, 285	Kaempferol 3-*O*-pentoside
33	31.42	583	169, 341, 431, 565	Isovitexin 2”-*O*-gallate
34	31.62	431	269	Apigenin 7-glucoside
35	33.34	447	151, 300, 315	Isorhamnetin 3-*O*-glucoside
36	33.61	461	299	Diosmetin-7-*O*-glucoside
37	34.26	583	179, 301, 431, 463	Quercetin 3-(6’’-*p*-hydroxybenzoylgalactoside)
38	35.52	625	179, 301, 463	Quercetin 3-*O*-(6’’-*O*-caffeoyl)-glucoside
39	38.08	567	255, 285, 447	Kaempferol 3-(6”-*p*-hydroxybenzoylgalactoside)
40	38.76	609	285, 323, 447	Kaempferol 3-*O*-(6’’-O-caffeoyl)-galactoside
41	39.98	567	255, 285, 447	Kaempferol 3-(6”-*p*-hydroxybenzoyl glucoside)
42	40.81	609	179, 301, 463	Quercetin 3-(3-p-coumaroylglucoside)
43	41.56	609	179, 301, 463	Quercetin 3-*O*-β-(6″-*O*-coumaroylglucoside)
44	42.85	301	151, 179, 301	Quercetin
45	44.57	593	285, 447	Kaempferol 3-(6′′-caffeoylglucoside)
46	45.62	593	285, 447	Kaempferol 3-(6′′-caffeoylrahmnoside)
47	50.18	269	107, 149, 269	Apigenin
48	52.30	285	151, 285	Kaempferol
49	57.21	439	163, 377, 395	*p*-coumaric acid derivative

^a^ Previously characterized from the stembark [19], ^b^ Previously described from the stembark [10].

### 3.2. Total Phenolic Content and Antioxidant Activity In Vitro

The extract revealed a total phenolic content of 274 mg GAE/g extract and demonstrated a considerable antioxidant potential compared to the reference compound, ascorbic acid (Table 3).

### 3.3. Effect of H. abyssinica Treatment on Passive Avoidance Behavioral Test in AD Rats

**Figure 2:**
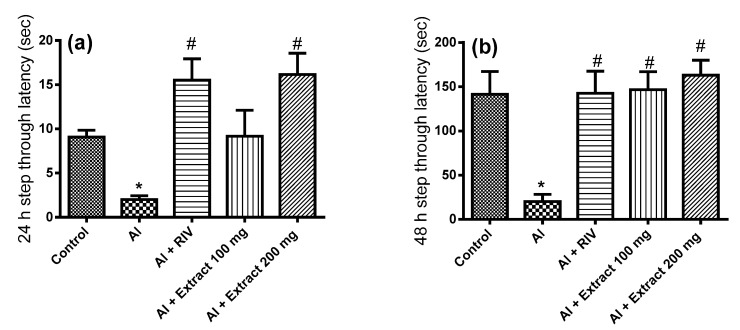
Effect of *H. abyssinica* extract on the passive avoidance behavioral test. The 24 (**a**) and 48 h (**b**) entrance latency to the dark compartment. * Significant difference from control group at *p* ˂ 0.001, ^#^ significant difference from AlCl_3_ group at *p* ˂ 0.001.

Changes in the behavior of the rats with AlCl_3_-induced AD were determined in a standardized “Passive avoidance test”. As shown in Figure 2, the entrance latency to the dark compartment 24 and 48 h after treatment declined in aluminum-treated rats compared to the control group. Administration of the AChE inhibitor rivastigmine (RIV) normalized the entrance latency. In the 24 h test, a low dose of the extract was not effective to restore normal entrance latency. However, a high dose of the extract restored the normal entrance latency at 24 and 48 h. In the 48 h test, all treatments restored retention latency in the illuminated compartment when compared to the AlCl_3_-treated group, with no significant difference between them.

### 3.4. Effect of H. abyssinica Treatment on AChE Activity, ERK and Glutamate Levels in the Hippocampus

**Figure 3:**
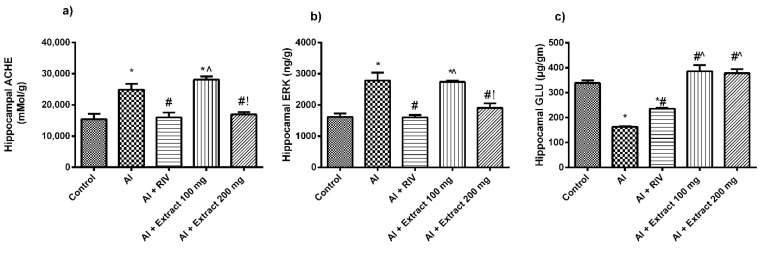
Effect of *H. abyssinica* extract on (**a**) AChE content and (**b**) ERK level in hippocampus. (**c**) Glutamate level in hippocampus. * Significant difference from control group at *p* ˂ 0.001, ^#^ significance difference from AlCl_3_ group at *p* ˂ 0.001. ^^^ Significant difference from RIV group at *p* ˂ 0.001, ^!^ significant difference from 100 mg group at *p* ˂ 0.001.

As shown in Figure 3a, the activity of AChE was significantly elevated in the hippocampus by 173% in the AlCl_3_-treated rats compared to the untreated rats. Treatment with either RIV or the extract at the high dose (but not the low dose) restored AChE activity in the AlCl_3_-treated rats. AlCl_3_ administration resulted also in a significant elevation of the hippocampal ERK level compared to the untreated rats. Treatment with either RIV or the high dose (but not the low dose) of the extract normalized the ERK level in the hippocampus of AD rats (Figure 3b). Additionally, AlCl_3_ administration significantly decreased the glutamate level in the hippocampus when compared to the untreated rats. Treatment with the extract (both doses) normalized the glutamate level in the hippocampus of AD rats with better activities than the reference drug (RIV) (Figure 3c).

### 3.5. Effect of H. abyssinica Treatment on MDA, TNF-α, IL-1β and Caspase-3 Levels

Some significant biochemical changes in the oxidative stress parameter MDA and the inflammatory biomarkers IL-1β, caspase-3 and TNF-α level were observed with the different treatments. In the AlCl_3_-treated rats, a significant increase in the brain MDA, IL-1β, caspase-3 and TNF-α levels was observed compared to the control group. On the other hand, AD rats treated with RIV showed an amelioration in the oxidative stress parameters and inflammatory biomarker levels when compared to the AD group. The AD group treated with the high dose of extract showed comparable results to those obtained from the RIV-treated group, and stronger response than that obtained from the group treated with the low dose of extract (Table 4).

### 3.6. Effect of H. abyssinica Treatment on Norepinephrine, Dopamine and Serotonin Levels

**Figure 4:**
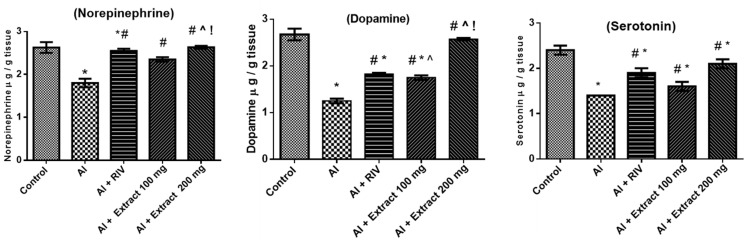
Effect of *H. abyssinica* extract on the levels of norepinephrine, dopamine and serotonin. * Significant difference from control group at *p* ˂ 0.05, ^#^ significant difference from AlCl_3_ group at *p* ˂ 0.001, ^^^ significant difference from RIV group at *p* ˂ 0.001, ^!^ significant difference from 100 mg group at *p* ˂ 0.05. Mean ± SE.

As shown in Figure 4, a significant decrease was apparent in the levels of the different neurotransmitters in the hippocampus of the aluminum-treated group compared to the control group. On the other hand, rivastigmine treatment increased norepinephrine, dopamine and serotonin levels when compared to the AD group. Both extract doses showed comparable effects to those obtained from the RIV-treated group when compared to the AlCl_3_-treated group (*p*-value ˂ 0.05). However, the effects of the extract’s high dose were more pronounced than those of the low dose.

### 3.7. Effect of H. abyssinica Treatment on mRNA Expression Levels of Bax and Bcl-2 Gene in the Hippocampus

**Figure 5:**
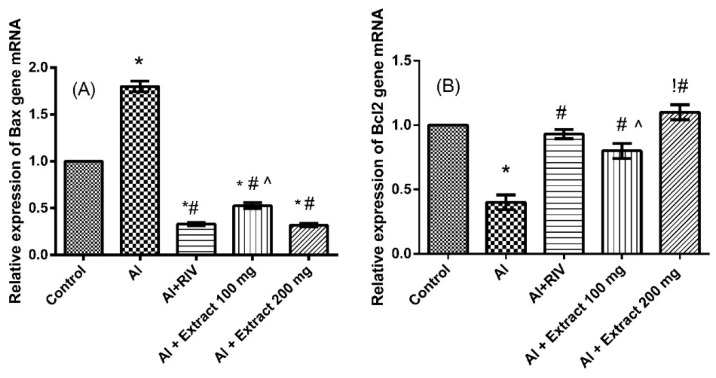
Effect of *H. abyssinica* extract on mRNA relative expression of (**A**) Bax and (**B**) Bcl2 in hippocampus. * Significant difference from control group at *p* ˂ 0.0001, ^#^ significant difference from AlCl_3_ group at *p* ˂ 0.0001, ^^^ significant difference from RIV group at *p* ˂ 0.001, ^!^ significant difference from 100 mg group at *p* ˂ 0.0001. Mean ± SE.

Induction of AD by aluminum treatment significantly decreased the pro-survival Bcl-2 (*p* < 0.0001) and increased the pro-apoptotic Bax (*p* < 0.0001) proteins compared to the control group (Figure 5). Bcl-2 expression was restored by the two doses of the extract (100 and 200 mg/kg). The extract treatments also downregulated the expression of Bax compared to the AlCl_3_-treated group. The high dose of the extract (200 mg/kg) showed comparable effects to the reference drug RIV.

### 3.8. Effect of H. abyssinica Treatment on the Histopathology of the Hippocampus

**Figure 6:**
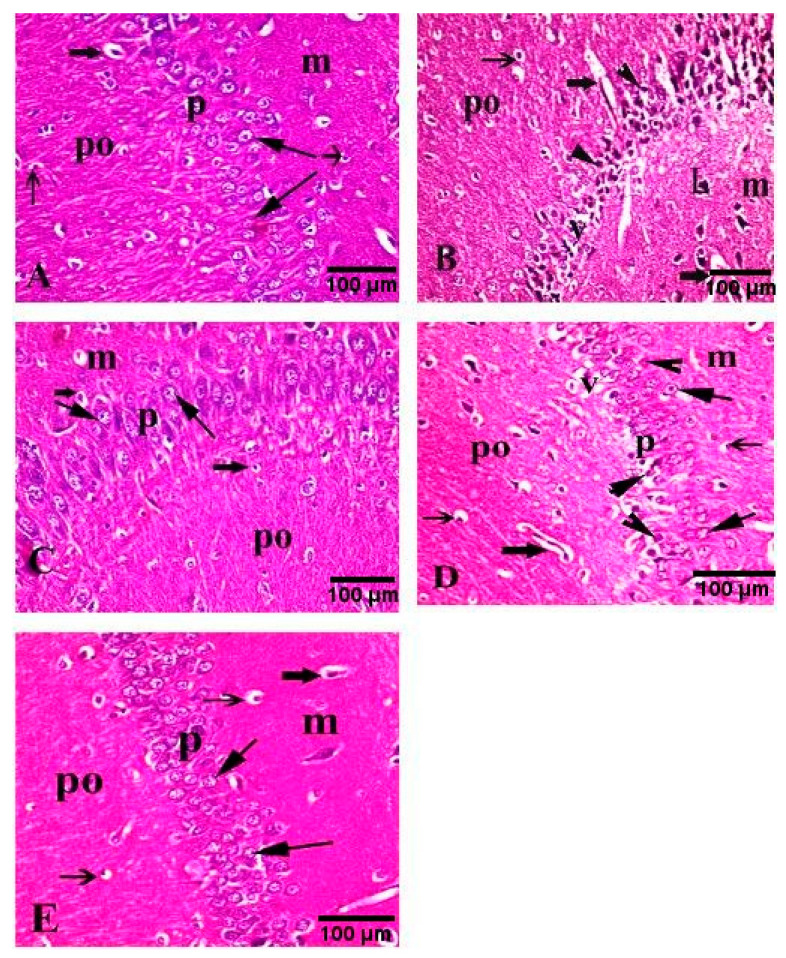
Photomicrographs of the hippocampal Cornu Ammonis region of rats (H&E, 400×). (**A**) Control rats revealed a normal appearance of molecular (m), pyramidal (p) and polymorphic layers (po). (**B**) AlCl_3_-treated animals revealed loss of normal structure. Distorted pyramidal layers reveal marked shrinkage and degenerative changes in pyramidal cells with pyknotic nuclei (arrowhead) and empty areas of pyramidal cell loss (v). Severe dilation of blood capillaries (thick arrow), enlargement of neurons (L) and glial cells (thin arrow) were noticed in molecular (m) and polymorphic layers (po). (**C**) AlCl_3_-rivastigmine-treated animals revealed normal histological structure of molecular (m), pyramidal (p) and polymorphic layers (po) with a mild reduction in pyramidal cells (arrow) of the pyramidal layer (p) and moderately dilated blood capillaries (thick arrow). (**D**) AlCl_3_/low dose of the extract-treated animals revealed some pyramidal neurons with normal structure, vesicular nuclei (arrow) and other neurons with moderate degenerative changes and pyknotic nuclei (head arrow) accompanied by loss of some cells (v). Mildly dilated blood capillaries (thick arrow) in polymorphic layer (po) and normal glial cells (thin arrow) in molecular layers (m) and polymorphic layer (po) were seen. (**E**) AlCl_3_/high dose of the extract-treated animals revealed normal appearance of molecular (m), pyramidal (m) and polymorphic layers (po). The pyramidal layer (p) showed reappearance of normal pyramidal cells with vesicular nuclei (arrow).

Microscopical examination of the hippocampal Cornu Ammonis region from the control animals revealed three layers; molecular layer, pyramidal layer and polymorphic layer. The pyramidal layer consisted of 4–5 compact layers of normal pyramidal neuron cells that appeared as triangular cells with large vesicular nuclei. Glial cells and blood capillaries were noticed in the molecular and polymorphic layer (Figure 6A). In contrast, brain sections from AlCl_3_-treated animals revealed loss of normal architecture, indicated by the distortion of pyramidal layers, extensive shrinkage and degenerative changes in pyramidal cells, along with the appearance of pyknotic nuclei and empty areas due to loss of pyramidal cells. Severely dilated blood capillaries, enlarged neurons and glial cells were noticed as well in the molecular and polymorphic layers (Figure 6B) compared to control group (Figure 6A). Regarding the rivastigmine-treated group, a normal histological structure of the three layers was observed with a mild reduction in the pyramidal cells (Figure 6C) compared to the AlCl_3_-treated group (Figure 6B). The low dose of extract-treated animals exhibited a moderate improvement revealed by the normal appearance of some pyramidal neurons with vesicular nuclei in the pyramidal layer and normal glial cells. Although some neurons still revealed moderate degeneration change with pyknotic nuclei. Loss of some pyramidal cells and mildly dilated blood capillaries were seen in the molecular and polymorphic layers. (Figure 6D) compared to AlCl_3_-treated group (Figure 6B). Animals treated with the high dose of the extract showed marked improvement, where no histopathological alterations could be found in the molecular, pyramidal and polymorphic layers in the Cornu Ammonis region (Figure 6E) compared to control (Figure 6A) and positive groups (Figure 6B).

### 3.9. Congo Red Staining of Hippocampus

**Figure 7:**
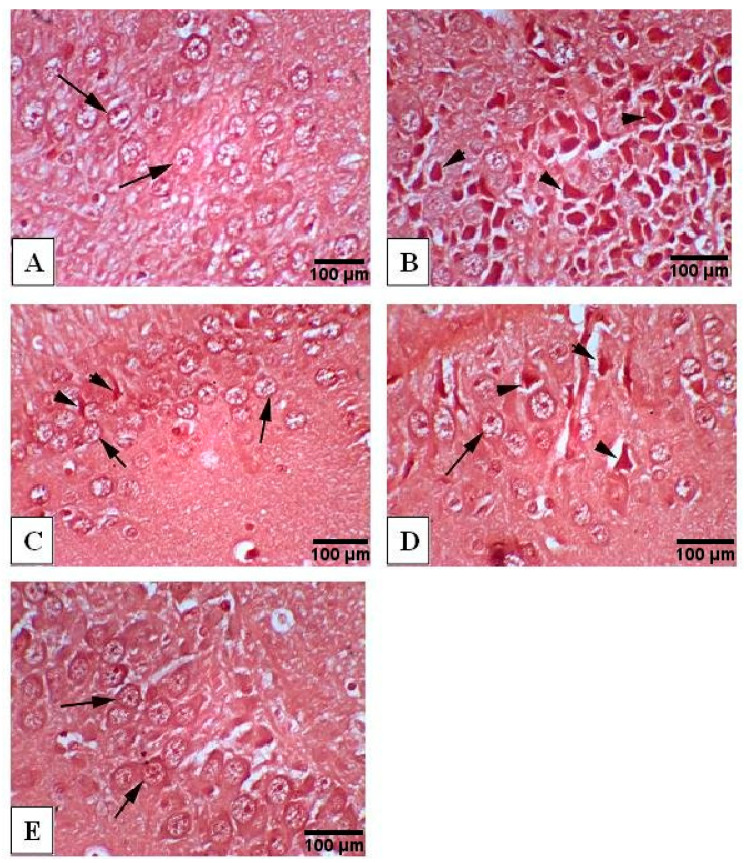
Photomicrographs of Congo red-stained hippocampus tissue (400×). (**A**) Normal group exhibiting normal histology structure of hippocampus with normal neuronal cells (arrow), (**B**) AlCl_3_-treated group exhibiting marked multifocal Aβ deposition in hippocampus (arrow head), (**C**) AlCl_3_/rivastigmine-treated group showing normal appearance of hippocampus with intact neuronal cells (arrow) combined with mild multifocal Aβ deposition (arrow head), (**D**) AlCl_3_/low dose of the extract-treated group showing normal histological appearance of hippocampus with intact neuronal cells (arrow) as well as mild to moderate multifocal Aβ deposition (arrow head), (**E**) AlCl_3_/high dose of the extract-treated group showing normal histological appearance of hippocampus with intact neuronal cells (arrow).

Hippocampus was examined after staining with Congo red dye to detect the Aβ deposition. AlCl_3_-treated animals (Figure 7B) demonstrated marked multifocal Aβ deposition in the hippocampus compared to the normal animals (Figure 7A). The hippocampus from AlCl_3_/rivastigmine-treated animals revealed mild deposition of amyloid beta plaques (Figure 7C) compared to the AlCl_3_-treated group. Treatment with the extract at the low dose (Figure 7D) revealed moderate deposition of amyloid beta plaques while treatment with the extract at the high dose (Figure 7E) revealed almost no deposition of amyloid beta plaques when compared to the AlCl_3_-treated group.

### 3.10. Molecular Docking

To gain some knowledge about the inhibitory potential of the individual extract’s components towards ERK2 and AChE, we docked the most abundant flavonoids into the two enzymes. The docked compounds perfectly fitted in the binding site of the two target enzymes and displayed minimum free binding energies. This is apparent upon comparing their docking scores (kcal/mol) relative to those of the natural and synthetic reference inhibitors used (Table 5).

Flavonoids identified in *H. abyssinica* showed appreciable free binding energy, indicated by low docking score values that ranged from −13.52 to −34.09 and −16.72 to −31.20 kcal/mol towards ERK2 and AChE enzymes, respectively. In general, all glucosides showed better docking scores and better binding energy to the target enzymes than the corresponding free aglycones (Table 5).

Bulkier glucosides with coumaroyl, caffeoyl or galloyl moieties afforded more hydrogen bonding and hydrophobic interactions with the amino acid residues in the binding sites of the two enzymes, and showed better binding energies relative to the lower-in-size glucosides bearing only 6- or 5-membered sugar moieties. Noteworthy is the fact that all the bulky glucosides were able to interact with the crucial Tyr337 amino acid in AChE and afforded the reported interactions with Ile31, Val39 and Met108 in ERK2.

**Figure 8:**
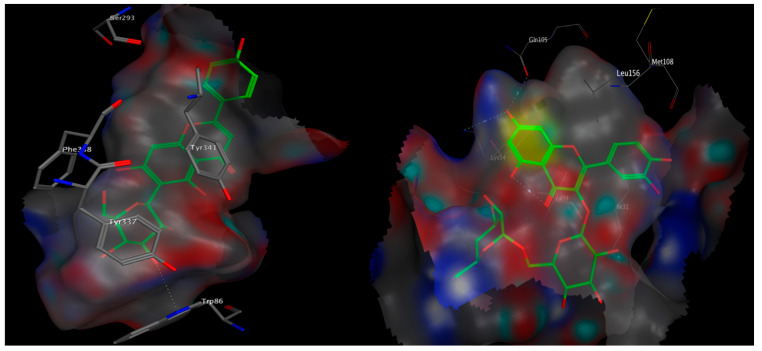
3D poses of apigenin 6-glucoside docked into the binding site of AChE (**left**) and quercetin 3-rutinoside docked into the binding site of ERK2 (**right**).

The C-flavonoid apigenin 6-glucoside, known as isovitexin, was the only small-in-size flavonoid that was able to reach out and interact with the Tyr337 residue in AChE with a comparable docking score (−26.97 kcal/mol) relative to that of the bulkier glucosides (Figure 8).

Quercetin 3-(6′-*p*-coumaroylglucoside) showed the best docking score and the utmost minimum binding energy of −31.20 kcal/mol towards AChE. On the other hand, quercetin 3-rutinoside (rutin) showed the best docking scores, the lowest binding energy (−34.09 kcal/mol) and the most reported amino acid interactions towards ERK2 (Figure 8).

## 4. Discussion

In the current study, we explored the behavioral changes induced by aluminum exposure in rats and the potential protective effects of a methanol extract from *H. abyssinica* leaves. The results of the present study confirm that chronic exposure to aluminum negatively influences the retention memory when evaluated by the passive avoidance test and histopathological examinations. Such an effect is probably due to the ability of aluminum to interfere with the downstream effector molecules, such as cyclic GMP, necessary for long-term potentiation, an interruption that could explain memory loss and neurobehavioral changes [20,21].

The cholinergic neurotransmitter acetylcholine (ACh) is vital for learning and memory [1]. The activity of the AChE is a well-renowned indicator for the damage of cholinergic neurons in the brain, as it is the primary enzyme inactivating ACh in the synaptic cleft [2]. Data from our study show that AlCl_3_ significantly enhanced AChE activity in the hippocampus of rats. The apparent increase in AChE activity by aluminum might be mediated by some allosteric interactions between Al^+3^ cation and the anionic sites of AChE in the brain [22]. Many AD treatments involve AChE inhibitors, such as rivastigmine or the alkaloid galantamine [23].

The signaling pathway involving the extracellular regulated kinase (ERK1/2) controls brain development and repair, synaptic plasticity and memory formation, and its overexpression is reported to be an essential factor in neuronal death and neuroinflammation [24]. Our obtained results show that the aluminum significantly enhanced ERK activity in the hippocampus of the AlCl_3_-treated rats.

Polyphenols, including flavonoids, are characterized by their substantial antioxidant potential. Several reports confirmed their ability to cross the blood–brain barrier and proved their potency in the treatment of Alzheimer’s disease via interfering with some potential targets, such as AChE and/or ERK2 [12,25]. For instance, the flavonoid quercetin 3-(2′,6′-diacetylglucoside) was shown to inhibit AChE with an IC_50_ of 36.47 µM [26]. The major polyphenol in green tea, epigallocatechin gallate (EGCG), was reported to inhibit the ERK signaling in the central nervous system and thus improved the brain function and prohibited oxidative stress and apoptosis [27]. In our study, treatment with the extract showed a reduction in AChE and ERK activities, which might be due to its phenolic and flavonoid content. As the experiments ran for 3 weeks, an influence on the expression of AChE and ERK is possible. Docking results confirm the potential of the major identified flavonoids in the extract to block the active site of both enzymes and afford the reported crucial amino acid interactions compared to the reference inhibitors.

From another perspective, aluminum can induce oxidative stress in the neurons, as it is an intoxicating pro-oxidant well known to augment lipid peroxides in the hippocampus [28]. This was notable in our study, as indicated by the significantly increased level of MDA in the AlCl_3_-treated rats. The administration of the extract significantly restored normal MDA levels. This positive outcome of the extract on oxidative stress is possibly due to its high content of antioxidant phenolics and flavonoids [12]. Polyphenols can protect rats from aluminum-induced brain neuroinflammation and cognitive impairments. Similar studies with polyphenol-rich extracts from pomegranate peel showed a reduced aluminum accumulation and stimulation of anti-apoptotic proteins in the rats’ brains [29,30].

With regard to Alzheimer’s disease, it is well substantiated that neuroinflammation plays a significant role in the development and pathogenesis of the disease. A wide spectrum of pro-inflammatory cytokines, such as TNF-α, IL-1β and caspase-3, are involved in neurodegeneration and apoptosis [31,32]. In our study, treatment with the extract exhibited a reduction in the proinflammatory cytokines and Aβ deposition with restoring normal histological appearance of hippocampus when compared to the AlCl_3_-treated rats. This is likely due to its flavonoid and phenolic content, which can protect neurons against neuroinflammation and neurotoxin-induced damage [33,34]. Moreover, the extract’s two dose levels were able to restore the normal expression of the pro-survival Bcl2 protein and downregulate the pro-apoptotic Bax protein.

Reduced levels of neurotransmitters belonging to the biogenic amine family in the brain is connected with a decline in memory in aging people [35]. In our study, AlCl_3_ significantly reduced noradrenaline, dopamine and serotonin levels in rat brains, in agreement with Kinawy et al. [36] and Foster et al. [37]. The treatment of rats with the extract significantly increased all monoamine levels. This is attributed to the presence of terpenoids, flavonoids and polyphenols, which have anti-inflammatory and antioxidant properties [38,39]. In summary, plant drugs rich with antioxidants have neuroprotective and anti-neurodegenerative roles by protecting against neuronal cell damage [40]. They offer a favorable potential in the control and management of numerous neurodegenerative conditions such as Morbus Alzheimer, Parkinson’s and Huntington disease [41,42,43].

## 5. Conclusions

This study proposes that the leaf extract from *H. abyssinica* normalizes catecholamine, ERK, caspase-3, AChE and glutamate content in the hippocampus of AD rats, accompanied by antioxidant, anti-inflammatory and antiapoptotic effects. It restored the original appearance of the hippocampus region of brain tissue as well, and was able to prevent the deposition of amyloid β plaques. These activities are most likely due to the high content of polyphenols. It could be thus concluded that *H. abyssinica* is a promising potential candidate to alleviate aluminum-induced neurotoxicity in the hippocampus. Further studies are needed to confirm the efficacy of the plant in treating Alzheimer’s disease and other neurodegenerative disorders.

## Figures and Tables

**Table 1:** Primer sequence for RT-PCR.

	Sequence	Accession Number
Bax	Forward 5’GAACCATCATGGGCTGGACA3’ Reverse 5’TGAGGTTTATTGGCGCCTCC3’	XM_032915032.1
Bcl2	Forward 5’GAACTGGGGGAGGATTGTGG3’ Reverse 5’ACTTCACTTGTGGCCCAGAT3’	XM_034943915.1
GAPDH	Forward 5’GACAGTCAGCCGCATCTTCT3’ Reverse 5’GCGCCCAATACGACCAAATC3’	XM_003819132.3

**Table 3:** TPC and antioxidant activity of *H. abyssinica* extract in DPPH and FRAP assays.

Sample	DPPH	FRAP	TPC
	EC_50_, µg/mL	mM FeSO_4_/mg Extract	mg GAE/ g Extract
Extract	9.6	18.24	274
Ascorbic acid	2.92	-	-
Quercetin	-	24.04	-

GAE = gallic acid equivalent.

**Table 4:** Levels of MDA, IL-1β, caspase-3 and TNF-α in the different treated groups.

Parameters	TNF-α	Caspase-3	IL-1β	MDA
	mg/g Tissue	pg/g Tissue	ng/g Tissue	nmol/g Tissue
Control	1.5 ± 0.06	22.00 ± 3	6.00 ± 0.3	170 ± 1.0
AlCl_3_	2.6 ± 0.05 *	158 ± 3.5 *	11.5 ± 0.5 *	305 ± 2.0 *
AlCl_3_ + RIV	1.9 ± 0.02 ^#^	51.5 ± 2.5 ^#^	8.00 ± 0.17 ^#^	124.5 ± 3.5 ^#^
AlCl_3_ + Extract 100 mg	3.2 ± 0.15 ^#^*^!^	91.7 ± 2.2 *^#^^	8.80 ± 0.7 ^#^	210 ± 0.02 ^#^^
AlCl_3_ + Extract 200 mg	2.1 ± 0.12 ^#^	68 ± 2 *^#^	7.30 ± 1.05 ^#^	178 ± 1.8 ^#^

Mean ± SE, * significant difference from control group at *p* ˂ 0.001, ^#^ significant difference from AlCl_3_ group at *p* ˂ 0.001, ^^^ significant difference from RIV group at *p* ˂ 0.001, ^!^ significant difference from 100 mg group at *p* ˂ 0.05.

**Table 5:** Docking score values (kcal/mol) obtained upon docking *H. abyssinica* polyphenols into ERK2 and AChE.

Compound Name	Docking Score (kcal/mol)	
	ERK2	AchE
Quercetin	−18.09	−19.75 *
Quercetin 3-glucoside	−22.50	−29.45
Quercetin 3-xyloside	−25.24	−29.53
Quercetin 3-rutinoside	−34.09	−26.31
Quercetin 3-(6′-p-coumaroylglucoside)	−27.19	−31.20
Quercetin 3-(6′-caffeoylglucoside)	−27.85	−29.03
Quercetin 3-(6′-galloylglucoside)	−28.32	−21.01
Quercetin 3-(6′-p-hydroxybenzoylgalactoside)	−27.50	−21.16
Kaempferol	−16.74	−17.69 *
Kaempferol 3-glucoside	−22.47	−25.25
Kaempferol 3-xyloside	−22.54	−22.87
Kaempferol 3-(6′-caffeoylglucoside)	−27.37	−29.88
Kaempferol 3-(6′-galloylglucoside)	−27.36	−27.76
Kaempferol 3-(6′-p-hydroxybenzoylglucoside)	−29.19	−25.19
Apigenin	−13.52	−16.72
Apigenin 7-glucoside	−21.20	−26.56
Apigenin 6-glucoside	−19.42	−26.97
Apigenin 6-(2′-galloylglucoside)	−30.03	−30.86
Co-crystallized inhibitor	−17.10	−18.38
EGCG (natural reference inhibitor)	−22.52	
Quercetin 3-(2′,6′-diacetylglucoside) (natural reference inhibitor)		−33.01

* Reported before [12].

## Data Availability

All data are included within the manuscript.
